# Modeling inter-individual differences in ambulatory-based multimodal signals via metric learning: a case study of personalized well-being estimation of healthcare workers

**DOI:** 10.3389/fdgth.2023.1195795

**Published:** 2023-06-09

**Authors:** Projna Paromita, Karel Mundnich, Amrutha Nadarajan, Brandon M. Booth, Shrikanth S. Narayanan, Theodora Chaspari

**Affiliations:** ^^1^^ HUman Bio-Behavioral Signals Lab, Texas A & M University, College Station, TX, United States; ^^2^^ Signal Analysis and Interpretation Laboratory, University of Southern California, Los Angeles, CA, United States

**Keywords:** metric learning, Siamese neural network, healthcare workers, well-being, mental health, ambulatory monitoring

## Abstract

**Introduction:**

Intelligent ambulatory tracking can assist in the automatic detection of psychological and emotional states relevant to the mental health changes of professionals with high-stakes job responsibilities, such as healthcare workers. However, well-known differences in the variability of ambulatory data across individuals challenge many existing automated approaches seeking to learn a generalizable means of well-being estimation. This paper proposes a novel metric learning technique that improves the accuracy and generalizability of automated well-being estimation by reducing inter-individual variability while preserving the variability pertaining to the behavioral construct.

**Methods:**

The metric learning technique implemented in this paper entails learning a transformed multimodal feature space from pairwise similarity information between (dis)similar samples per participant via a Siamese neural network. Improved accuracy via personalization is further achieved by considering the trait characteristics of each individual as additional input to the metric learning models, as well as individual trait base cluster criteria to group participants followed by training a metric learning model for each group.

**Results:**

The outcomes of the proposed models demonstrate significant improvement over the other inter-individual variability reduction and deep neural baseline methods for stress, anxiety, positive affect, and negative affect.

**Discussion:**

This study lays the foundation for accurate estimation of psychological and emotional states in realistic and ambulatory environments leading to early diagnosis of mental health changes and enabling just-in-time adaptive interventions.

## 1. Introduction

Healthcare workers often experience significant job strain as a result of high physical, mental, and emotional workloads with low decision latitude and extensive responsibilities (1). These job characteristics can lead to high burnout rates, which can be the source of stress, anxiety, and depression (2). In light of the 2019 novel coronavirus (SARS-CoV-2) pandemic, commonly known as the COVID-19 pandemic, the importance of the well-being of healthcare workers has become a primary concern (3). There has been a wide array of interventions at the institutional and personal level attempting to combat burnout of healthcare workers (4). Many medical schools and hospitals have shifted their focus on institutional policies that promote healthy work conditions, including hiring additional staff for administrative duties, introducing counseling services, mental health awareness campaigns, mentoring meetings, and group discussions with faculty and staff (5–8). Apart from these, mindfulness training and social cohesion wellness programs (e.g., mandatory wellness retreats) have been particularly important in promoting personal and social well-being (9–14). Such initiatives can create safe spaces for healthcare workers to share their experiences and help hospital workers become more aware of their thoughts and feelings, thus effectively reducing the adverse effects of burnout and promoting a healthy and supportive work environment. Yet, there is no “one-size-fits-all” approach to these interventions, which rather need to be tailored to the population of interest and its corresponding capabilities and characteristics. Moreover, attending the aforementioned programs and training sessions after long work hours often becomes burdensome for healthcare workers, rendering these initiatives ineffective in many cases.

Just-in-time adaptive interventions (JITAIs) are an emerging new intervention design for mental health that aims to provide the right type and amount of support at the right time, depending on a user’s emotional state and other contextual factors (15–17). JITAIs leverage ambulatory devices, such as smartphones and wearable sensors, offering the means to track the moods and emotions of healthcare professionals in a personalized manner. They can assess one’s behavior in a manner that is often inaccessible in standard clinical settings, providing ecologically valid data of complex cognitive, affective, and psychological processes over time for a particular individual, and potentially contributing to the early detection of health degradation (18, 19). Ambulatory data can provide a foundation for personalized interventions for healthcare workers that can potentially mitigate negative mental health outcomes, augment their work performance, and improve the overall care received by the patients in the hospital (20). Ambulatory data can further offer an alternative to self-reports (21), which are prone to response bias and recall bias, often resulting in under-reporting or over-reporting symptoms (22). Previous studies indicate that with proper prior testing and troubleshooting of the deployed ambulatory systems, healthcare workers overall respond positively to utilizing ambulatory sensors for monitoring wellness (23).

Several challenges affect ambulatory monitoring in the wild. Ambulatory data are collected in uncontrolled real-world environments and might be affected by various confounding factors, such as co-occurring activities (e.g., exercise), interpersonal relationships (e.g., conflict with co-workers), and environmental conditions (e.g., indoor ventilation, outdoor weather conditions) (24). The signals recorded by ambulatory sensors are further prone to non-standardized factors in the data collection process, including sensor misplacement and non-fixed physiological baseline (25), which result in highly variable data distributions (26). Finally, inherent individual differences (e.g., in terms of demography and psychological factors) often influence the collected human behavioral data and can be manifested across various signal modalities (27, 28). These impose significant challenges to the design of machine learning methodologies that can reliably quantify facets of psychological and emotional outcomes from ambulatory data.

We may address the inherent individual differences in signals collected through ambulatory sensors by constructing models that reduce the evidence of person-specific information on the data while preserving information about the behavioral outcome of interest (29). Toward this end, personalized and group-specific models have recently been the focus of multiple research efforts, since they provide tailored estimates of behavioral constructs for each participant or group of participants. Personalized models are tailored to a specific participant via learning their individual behavioral patterns (30), as well as integrating their demographic and anthropomorphic characteristics (31, 32). Such approaches usually involve the learning of separate models for each participant (33), which entails the risk of over-fitting or inadequate training due to the lack of enough data from a given subject. Group-specific models cluster participants according to clinically and theoretically relevant characteristics and are usually implemented through hierarchical and adaptive learning methods (34–36). Such methods use a portion of data from a target entity to fine-tune the decisions of a machine learning model adaptively (37, 38). Increasing the amount of target data used for fine-tuning the models tends to improve their performance (39). Metric learning techniques, such as Siamese neural networks (SNN), require a small amount of data to fine-tune such models as they model the relative distance between samples, rather than the absolute class-wise patterns (40). Thus, metric learning methods are a promising solution to personalized and group-specific models when the labeled samples from a target entity are scarce.

In this study, we propose a metric learning technique for estimating the well-being of healthcare workers in a personalized manner. Metric learning is implemented with an SNN architecture that learns a multimodal signal transformation that models the pairwise similarity between similar and dissimilar samples from each participant. The proposed pairwise similarity approach does not require the separate estimation of the data distribution for each class, therefore it can be more robust to outliers and more forgiving to small datasets (41). We have further examined the effect of including an increasing number of labeled samples from the target participants to fine-tune the SNN. Finally, as an additional personalization step, we have integrated participants’ trait characteristics in two ways: (1) as an input to the models; and (2) as a clustering criterion to group participants, followed by training separate models for each group. Our proposed approach is evaluated on a dataset collected in a real-world hospital environment over time, including data from 139 healthcare workers with constructs related to well-being (i.e., anxiety, stress, positive/negative affect) (42). Our findings suggest that the proposed approach preserves the variability attributed to the behavioral outcome of interest yielding promising results in terms of estimating behavioral constructs while minimizing user-dependent variability. The proposed metric learning approach further outperforms non-personalized models, such as a feedforward neural network (FNN) trained on all participants using the original ambulatory-based features, as well as conventional distribution-based adaptation methods, such as the maximum independence domain adaptation (MIDA), that attempts to mitigate inter-individual differences by aligning feature distributions from all participants. Implications of this study result in the accurate longitudinal tracking of well-being in highly demanding job settings and help lay a foundation to apply JITAIs for mental health (17).

## 2. Prior work

Ambulatory devices can unobtrusively collect data from participants in their daily lives, providing us with informative insights regarding daily activities, physical outcomes, and behavior patterns, therefore informing treatment and intervention procedures (43, 44). Smartphones are widely used devices that provide an effective way to quantify behavior via the various in-built sensors, while at the same time integrating the computational power to analyze the signals captured by the sensors in a meaningful manner (45). In a study conducted by Hunasgi et al. (46) aiming to recognize stress in dental students, an Android smartphone app called “S-HEALTH” has been used to detect heart rate, oxygen saturation, and stress levels via smartphone sensors. The authors collected ambulatory-based data before and after a stressful event, during which physiological recordings have been collected by asking participants to touch a specific sensor of the smartphone device. Apart from collecting physiological data, smartphones have been also used to collect other behavioral metrics, such as user feedback data, overall phone activity, speech, location, and physical movement, which play a key part in detecting stress (47–49), anxiety (50), negative affect (51), unusual activities (52), and mood (53). Wearable devices can further enable physiological recording in a real-world setting (i.e., “in-the-wild”). Data collected through activity trackers, such as Fitbit (54), have been used to predict health and wellbeing status (55), detect daily stressful events (56) or predict future mood (57–59). Schmid et al. have used wearable sensors to track healthcare workers’ heart rate variability (HRV) during mindfulness exercises (60). Sano et al. have used wrist-based sensors to record the physical activity and autonomic physiology of college students over the span of a month with the goal of assessing stress and mental health (61). Smartphones in combination with wearable devices have been used in several other studies to detect stress and depression (47, 62, 63). Gaballah et al. proposed a context-aware speech stress detection model using the TILES-2018 dataset (42), which is the same dataset we have used in our study. Gaballah et al.’s study utilizes audio, location, and circadian rhythm signals extracted from different ambulatory devices, for example, smartphones, smartwatches, and smart garments, from 144 healthcare workers working for 10 weeks to detect stress in the wild. Their proposed bidirectional long short-term memory (LSTM) model reached a classification F1 score of up to 65%, while environmental sensor data acted as the context for the model (64). Another study uses physiological, physical, sleep, and context features from TILES-2018 and the TILES-2019 datasets (65) to develop personalized models to predict future affect. They achieve personalization by developing individual-specific random forest and mixed-effect random forest models. These models were trained using the data collected per individual during the first 2–8 weeks of the total 10-week data collection period and predicted future affect reaching correlation coefficients up to 0.8 for positive affect and 0.5 for negative affect (66). Multi-modal signals collected using an assortment of ambulatory devices from a large (N=606) number of individuals, were used to detect stress in the wild in a separate study. Through a regression analysis, this study reached a Spearman correlation of 0.25 using a combination of random forest and state-trait anxiety inventory to detect stress (67).

The data collected through ambulatory devices often demonstrates high-inter individual variability, which can be addressed via the design of personalized models that can accurately model physical and behavioral outcomes while at the same time eliminating the evidence of inter-individual variability in the data attributed to behaviorally non-relevant information (51, 68, 69). Personalized machine learning models are popular in various domains, such as in computer vision where models have been used to capture the inherent individual diversity in detecting facial expressions and gestures from unlabeled data (37) or to generate automatic tags for a video based on a user’s comments and preferences (70). Personalized models implemented with multitask learning have been also employed to recognize human activity from multimodal sensor data (71, 72) and in the detection of unusual events pertaining to a user’s activity using speech data recorded via smartphone devices (52). Similarly, De Santos et al. have utilized personalized models to increase bio-metric system security via detecting deviation from normal behavior for accurate person identification (73). While studying the impact of meal consumption on one’s glucose response, Zeevi et al. have opted to use personalized models given the high inter-individual variability of the glucose response signal (69). Personalized models have been also employed to develop treatment regimens, where such models have been trained to optimize a target criterion, such as maintaining blood glucose at a healthy level (74, 75). They have been further utilized to detect momentary negative affect of undergraduate students via incorporating contextual data (e.g., location, movement, phone usage) collected from smartphone devices (51), and have been found to outperform generalized models for the same task (58, 76).

Personalized models may be developed following various procedures, for example, via modeling data separately for each individual (30), via transfer learning (by fine-tuning a general model using data from a target individual) (37), or via integrating individual factors to the model input, such as anthropomorphic, psychological, and cognitive characteristics (32, 69). Psychological studies have found several individual factors, such as coping styles and current life conditions (e.g., poor family relationships), to be related to stress moderation, and physiological and affective responses (77–79), motivating the integration of such factors into the design of personalized machine learning models. A relatively small number of studies have considered individuals’ trait characteristics when modeling behavioral outcomes. Gupta et al. and Yadav et al. have leveraged individuals’ trait characteristics, such as trait anxiety, personality, and attachment variables, to form clusters of participants, based on which they have designed population-specific models of behavior (36, 80). In supporting sleep health, Nguyen et al. have utilized personality traits and chronotype characteristics (i.e., one’s morning or evening preference) to develop personalized feedback for sleep interventions (81). Tondello et al. have further used five gaming traits that reflect participants’ gaming preferences in developing a personalized gaming platform (82). Individual characteristics, such as ideology, dominant emotional sentiments, and personality have been further utilized to develop a personalized psychological intervention system to promote inter-group relations (83). This evidence suggests that considering individuals’ characteristics is an important design factor toward personalized behavior detection models and subsequently, effective JITAIs.

Data collected from ambulatory devices are prone to errors related to ambient noise and sensor misplacement. In addition, it is usually difficult to obtain labels from all samples during ambulatory monitoring studies, therefore labeled data are not always sufficient for modeling absolute patterns in data distributions for each participant (24). Few-shot learning techniques that are commonly based on metric-learning approaches model the relative distance between samples, thus can be trained with a small amount of data (40). These models have the ability to naturally rank data samples based on sample pair similarity and have shown promising results in image recognition (84), visual tracking (85), and detection of the continuous spectrum of disease severity (86). Siamese neural networks (SNNs) have also found applications in speech-based emotion recognition (87, 88) and gait-based user identification (89). Although SNNs have been employed extensively for classification purposes, regression analysis using SNNs is a field that is not extensively studied (90). Moreover, none of the previous works have formulated such techniques in the context of personalized learning of behavioral outcomes.

The contributions of this paper are the following: (1) we introduce a novel metric learning approach implemented with an SNN regressor to learn personalized representations of multimodal signals and their association with behavioral outcomes; (2) we investigate additional ways to personalize our models by utilizing the participants’ trait characteristics and fine-tune the models using a portion of the data collected from the target participants; and (3) we evaluate these methods on ecologically valid data collected in situ from healthcare professionals with variable job responsibilities and high-pace work conditions.

## 3. Data description

Our data is part of the TILES-2018 dataset (42), which was collected from 212 full-time hospital workers, aged between 21–65 years, over 10 weeks. Participants engaged in their typical daily activities while being equipped with ambulatory wearable devices and sensors to collect vocal acoustic and physiological signals throughout the period of data collection. A Fitbit Charge 2 was used to measure sleep activity and exercise, an OMsignal garment collected heart rate and breathing rate, and the Unihertz Jelly Pro smartphone, a small and lightweight phone worn on the lapel, was programmed to obtain vocal acoustic features from statistically sampled egocentric audio recordings (91).

Participants completed several surveys during the enrollment and data collection period. Initial ground truth battery (IGTB) surveys were collected once during the enrollment period to assess participants’ trait characteristics related to job performance, cognitive abilities, and health. Cognitive IGTB constructs include participants’ organization citizenship behaviors (OCB), which were measured using the Organization Citizenship Behavior Checklist (OCB-C) (92) via 20 items on a scale ranging from 1–5, and cognitive ability, which was captured using both the Shipley Abstraction Test (93) assessing fluid intelligence (25 items on a scale of 0–25) and the Shipley Vocabulary test assessing crystallized intelligence. In the Shipley Vocabulary test, participants would match words with their synonyms from a list of 40 items, and the score is calculated from the correctness of the matching between the words. Psychological IGTB constructs include personality traits (i.e., extraversion, agreeableness, conscientiousness, emotional stability, openness), assessed via the Big Five Inventory-2 (BFI-2) (94), trait affect (i.e., the participants’ overall affect that does not depend on momentary factors) captured with the Positive and Negative Affect Schedule Expanded form (PANAS-X) (95) using 60 items on a scale of 1–5, and trait anxiety measured by the Trait-Form of the State Trait Anxiety Inventory (STAI) (96). Apart from this, we have utilized the tobacco usage data, collected through the 3-item Global Adult Tobacco Survey (GATS) (97) and sleep quality data, collected through the 19-item Pittsburgh Sleep Quality Index (PSQI) (98) for our experiments. The IGTB measures are used as an additional input to the machine learning models, as well as a clustering criterion to group participants, followed by training separate models for each group.

**Figure 1:**
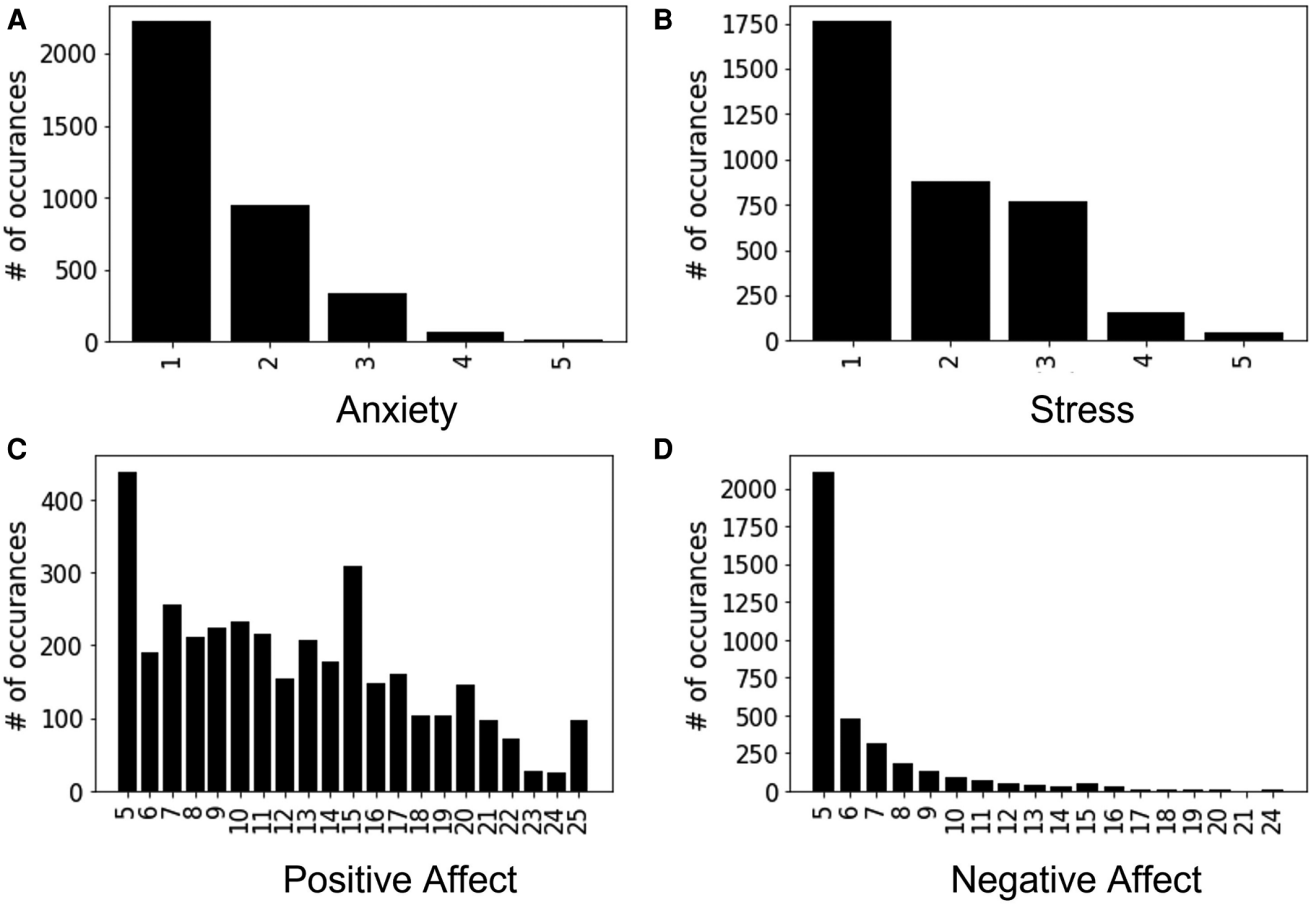
Distribution of momentary ground truth (MGT) data used as outcomes.

Momentary ground truth (MGT) surveys of well-being constructs were collected once per day through a smartphone app using ecological momentary assessments (EMAs) related to participants’ anxiety, stress, positive affect, and negative affect. To make the daily self-reporting more accessible to the participants, the number of assessment items is reduced while following the prior standard scaling format with significant reliability (42). Daily measurement of anxiety and stress was conducted by asking two questions (i.e., “Please select the response that shows how anxious you feel at the moment” and “Overall, how would you rate your current level of stress?”) and were both assessed on a 5-point Likert scale. Affect was obtained through the Positive and Negative Affect Schedule-Short form (PANAS-S) (99), which includes 10 items, 5 items each for positive and negative affect. Each item of this questionnaire was scaled from 1 (very slightly or not at all) to 5 (extremely), resulting in a label value ranging between 5–25. This study uses the MGT constructs of anxiety, stress, positive affect, and negative affect as the behavioral constructs of interest, that represent participants’ well-being outcomes. The corresponding distributions of these constructs are presented in Figure 1, where each x-tick represents the discrete values of the construct (e.g., self-reported anxiety MGT is labeled between 1–5, thus there are 5 representations in Figure 1A) and y-tick represents the total count of some particular label present in the full dataset.

Much effort was spent before and during the data collection to maximize the quality and ecological validity of the data (100). Despite this effort, some of the participants had missing data from one or more sensors as well as missing ground truth information from the self-reports. For this study, 73 participants had less than 13 days of self-reported data out of the 10-week study period, where 13 is an empirically chosen minimum number of days for which participants require self-reports. Since self-reports act as a ground truth for the proposed machine learning methods and proper ground truth information is necessary for supervised learning, we removed these participants from the total pool, resulting in 139 participants with on average 3-week data that were included in the experiments. From these 139 participants, around 10% of feature values on average per participant were missing. These feature values served as the input to the machine learning models and were replaced using linear interpolation Section 4.1.

## 4. Methodology

This section delves into details of processing the data, designing the proposed models, and evaluating our approach. Section 4.1 contains details on how we pre-processed our data and extracted features. The loss functions to achieve personalization through metric learning and the models developed for different experiments are documented in the Section 4.2. Section 4.3 describes the details of our experimental framework and Section 4.4 refers to the baselines used to compare the performance of our proposed models.

### 4.1. Data pre-processing and feature extraction

**Figure 2:**
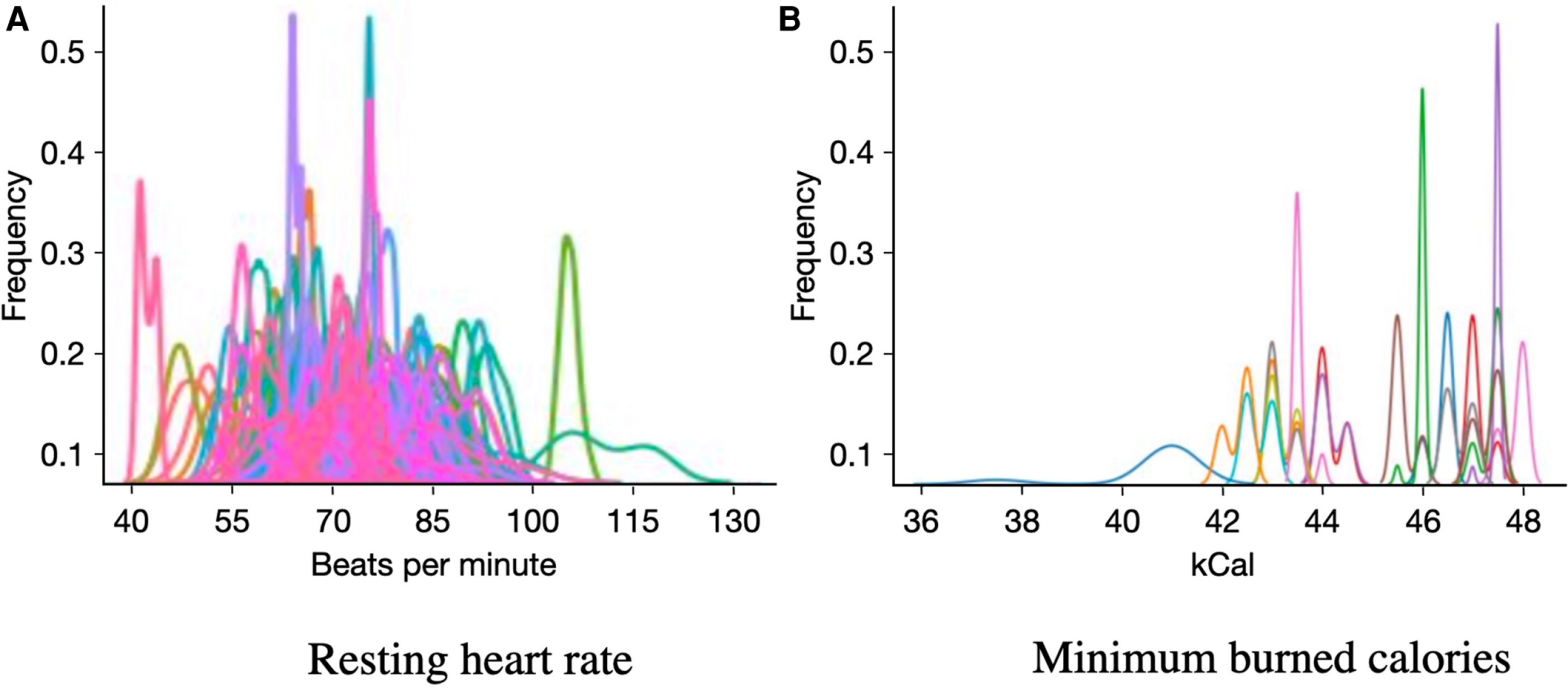
Histograms of feature distributions measured from the ambulatory data color-coded by participants.

The input features of the different models included both ambulatory measures and participants’ trait characteristics. We used 69 ambulatory features available as part of TILES-2018 dataset, which include 25 measures of physiology and daily activity from Fitbit Charge 2, 29 acoustic features from Unihertz Jelly Pro, and 15 physiological features from OMsignal garment (42) (Table 1). We accounted for the missing values at the sample level of the features by replacing the missing data points through linear interpolation using the data points before and after the missing data. We further calculated the daily average of these features for our analysis. Figure 2 depicts the distribution of two example features, namely, the resting heart rate and the minimum number of burned calories, for each participant. We observe large inter-individual differences in these features’ range and distribution shape, providing evidence of the need to design personalized models.

**Table 1:** Description of ambulatory features recorded continuously over 10 weeks via wearable devices.

Device	Feature description	# Features
Fitbit charge 2	Upper/lower threshold of cardio activity range (CAR)${}^{*}$/fat burn activity range (FBAR)${}^{*}$/peak activity range (PAR)${}^{*}$/out of zone activity range (OORAR)${}^{*}$, number of minutes and calories burned in CAR/FBAR/PAR/OORAR, number of steps, minutes awake, minutes in deep/light/REM/non-REM sleep, minutes asleep, minutes in bed, sleep efficiency	25
Unihertz Jelly Pro	Jitter, jitter 1st-order derivative, shimmer, fundamental frequency (average, average of smoothed contour, average of smoothed contour envelope), harmonic-to-noise ratio, voice probability, signal norm, signal norm computed with RelAtive SpecTrAl (RASTA)-Perceptual Linear Predictive (PLP) methodology, energy, zero-crossing rate, intensity, loudness, Fast Fourier transform (FFT) magnitude (250–650, 1000–4000 Hz), spectral roll-off of interquartile range (0–25%, 25–50%, 50–75%, 75–90%), FFT magnitude spectral flux, FFT magnitude spectral centroid/entropy/variance/skewness/kurtosis/slope, FFT magnitude sharpness/harmonicity	29
OMsignal garment	Breathing rate, heart rate, intensity, average heart rate, average X/Y/Z acceleration, root mean square of first R-R interval difference, total power, very low/low/high frequency power, low to high frequency power	15

${}^{*}$OORAR/FBAR/CAR/PAR/: 0–50/50–69/70–85/85–100% of maximum heart rate.

Furthermore, as an additional input to the proposed personalized models, we have considered 14 IGTB features that are indicative of participants’ trait characteristics, as described in Section 3 and summarized in Table 2. All the samples used in our experiments are independent over time. Also, we conducted min-max feature normalization to these ambulatory features using the minimum/maximum value of the corresponding training set’s participants to ensure uniformity while using them for training our proposed models.

**Table 2:** Description of initial ground truth battery (IGTB) features collected once during the enrollment period.

IGTB type	IGTB description	# features
Cognitive	Organization citizenship behaviors, fluid intelligence, crystallized intelligence	3
Psychological	Extraversion, agreeableness, conscientiousness, emotional stability, openness, trait positive affect, trait negative affect, trait anxiety	8
Health	Tobacco use (Yes/No), Tobacco quantity, sleep quality	3

### 4.2. Metric learning for personalized models

The metric learning approach, which we implement via SNN, learns the relative distance between two items in a pair based on whether the items are similar or dissimilar. So, we form pairs of samples to train our proposed model. The pairs could be formed using two similar or dissimilar instances of input (x) as described in Section 4.2.1. One of the ways we achieve personalization in this study is by rigorously following a participant-wise format while forming the pairs. The metric learning is achieved via equation 1. Here we calculate the relative distance between the transformed embeddings ($f_{W}(x_{nm})$ and $f_{W}(x_{nm^{\prime }})$) and the normalized ground truths (${\hat{y}}_{nm}$ and ${\hat{y}}_{nm^{\prime }}$) among the pairs we formed for our SNN structure. Subsequently, the transformed embedding ($f_{W}(x_{nm})$) is used in the regression analysis, where through another layer of transformation ($g_{V}$), the learned embedding helps in predicting the constructs considered in this study. The regression loss, described in equation 2, helps in updating the whole structure for better performance. In the following, we will describe the metric learning formulation that has been used to implement the proposed personalized models.

Let $D_{n}=\{x_{nm},y_{nm}\}$ be the set of data obtained from participant n, where $x_{nm}$ and $y_{nm}$ represent the feature vector and MGT label of the mth sample from participant n. Let ${\hat{y}}_{nm}$ represent the $\ell_{2}$-normalized version of $y_{nm}$. Metric learning learns a transformation (or embedding) $f_{W}$ of the input feature, parameterized through W, such that pairs of samples corresponding to proximal levels of the MGT outcomes are projected to the same neighborhood of the feature embedding, while the opposite occurs for samples with a distinctively large difference in the MGT outcome. This is implemented via the following loss function:

(1) $$l_{F}=\sum_{n}\sum_{m\neq m^{\prime }}\left|(\|f_{W}(x_{nm})-f_{W}(x_{nm^{\prime }}){\|}_{2}-\|{\hat{y}}_{nm}-{\hat{y}}_{nm^{\prime }}{\|}_{2})\right|$$

where (1) defines the difference between the distance of samples m and $m^{\prime }$ of participant n with respect to the transformed feature space $f_{W}$ and the MGT label space. The distance with respect to both the embedding and label space is measured via the Euclidean distance, where $\|\cdot {\|}_{2}$ represents the $\ell_{2}$-norm.

In order to impose additional supervision and achieve a reliable estimation of the MGT outcomes, the feature embedding $f_{W}$ is further transformed to the final MGT outcome via $g_{V}$, parameterized through weights V. The parameters included in V are learned using the following loss function, which minimizes the mean square error between the actual and estimated MGT output:

(2) $$l_{R}=\sum_{n}\sum_{m}\|g_{V}(f_{W}(x_{nm}))-y_{nm}{\|}_{2}^{2}$$

**Figure 3:**
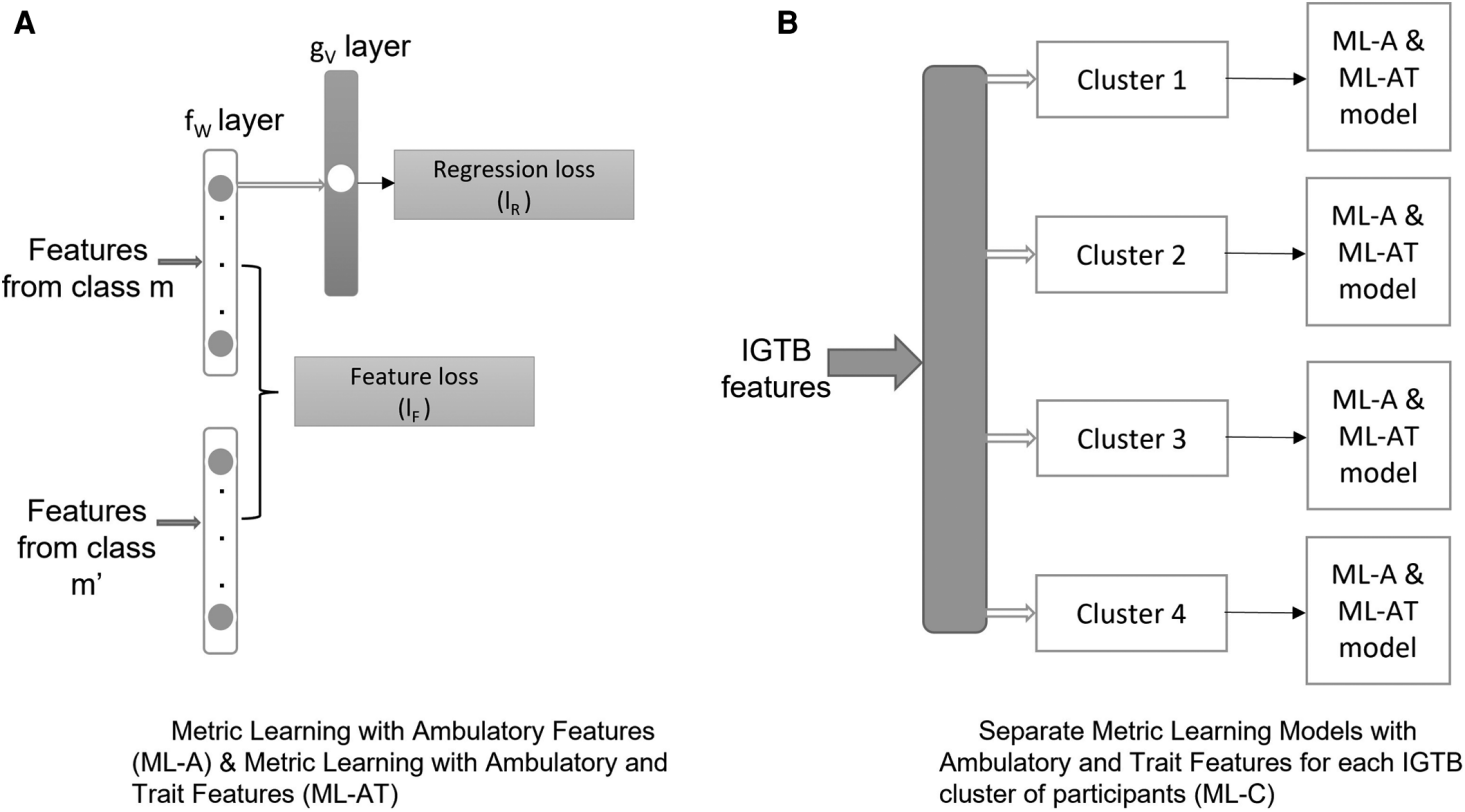
Schematic representation of metric learning implemented with Siamese neural networks.

The above optimization problem can be implemented via an SNN (Figure 3A). The SNN takes as an input pair of samples from the same participant and projects those into a feature embedding via the transformation $f_{W}$ in the shared $f_{W}$ layer of the schematic diagram (Figure 3), which minimizes the feature loss $l_{F}$. The output of the SNN is an MGT outcome resulting from transforming the feature embedding via the transformation $g_{V}$, which minimizes the regression loss $l_{R}$.

Utilizing this formulation, we conducted an ablation study comprising three models: (1) metric learning with ambulatory features (ML-A): this model takes the ambulatory features as input to the model (Section 4.2.1), (2) metric learning with ambulatory and trait features (ML-AT): this model takes IGTB features representing participants’ trait characteristics, along with the ambulatory features as input to the model (Section 4.2.1), and (3) separate metric learning models with ambulatory and trait features for each IGTB cluster of participants (ML-C): after developing clusters of data based on IGTB values, this model applies the ML-AT model on separate clusters (Section 4.2.2). A detailed description of each of these is provided below.

#### 4.2.1. Metric learning with ambulatory features (ML-A)

As the first model of our ablation study, ML-A incorporates personalization through personalized pair formation (Section 4.2) and transfer learning. We study this model to understand how personalized models using only the ambulatory features performs in predicting different job constructs. ML-A was implemented with an SNN whose input includes the 69 ambulatory features described in Table 1. For training the SNN, the original dataset was divided into 10 stratified folds with each fold containing around 14 participants. We conducted the “participant-independent cross-validation” process for training and testing our models, thus there was no participant overlap across folds. Among the folds we developed, one fold is utilized for validation, one fold for testing, and the remaining were used to train the model in a looping fashion ensuring we have tested all the folds using separate models. We ensured that no overlap exists among training, validation, and testing folds, thus allowing us to explore the generalizability of the models to unseen samples. Moreover, all the model parameters were removed after each loop for testing each of the folds to avoid data leakage.

In order to form the pairs to train the model, we defined two labels of the instances to form the pair as being similar if |a−b|≤1∀a,b∈ *labels*, and the rest dissimilar given the condition is not met empirically. Although positive affect and negative affect have a considerably different range of labels than anxiety and stress, outcomes of experimentations, while changing the threshold for similarity from 1 to 5, did not show a significant difference in performance. Moreover, this has helped us tackle the problem of the four target constructs having different ranges of labels (anxiety: 1–5, stress: 1–5, positive affect 5–25, negative affect: 5–24).

To further personalize our model, we utilized the transfer learning process by taking 0–90% of testing data (i.e., including a percentage of samples between 0–90% from the target participants in the test fold), referred to as “fine-tuning data,” and included that in training fold for the purpose of fine-tuning our model. This part was done after the hyper-parameters were set via the hyper-parameter tuning process (explained in Section 4.3) for the given fold and at the beginning of the SNN training. We removed the “fine-tuning data” from the target participants’ data of the testing folds to ensure that any data leakage is completely avoided during the training and testing of the models. Algorithm 1 outlines the training process of the ML-A method. It depicts one single iteration of the algorithm which has the outer loop looping through F folds. Inside the outer loop, there are two separate loops, the first loop helps in preparing the pairs, and the second loop trains the model until the necessary condition for the early stopping is met. With random initialization, we run this algorithm 20 times for this model.

**Algorithm 1:** Algorithm Describing the Metric Learning with Ambulatory Features (ML-A) model and theMetric Learning with Ambulatory and Trait features (ML-AT) model.

**Input = folds** (F), **participants** (N), **features** *X* **and labels** (Y) **Output = Predicted label** $\left(\bar{Y}\right)$ 1: {Creating folds from participants, one fold for testing, one fold for validation, remaining for training. Samples are taken from the training set. Hyper-parameter tuning and early stopping are done using the validation set. During each for loop for fold, testing is done.} 2: **for** f←1toF **do** 3: **for** n←1toN **do** 4: ${\;x}_{nm},{\;x}_{nm^{\prime }}\leftarrow \;formed\;pairs\;within\;each\;participant\;(similar/dissimilar)\;with\;m\;and\;m^{\prime }\;samples$ $\text{where}\;m\neq m^{\prime }$ 5: $y_{nm},y_{nm^{\prime }}\leftarrow original\;labels\;for\;m\;and\;m^{\prime }\;samples$ 6: ${\hat{y}}_{nm},{\hat{y}}_{nm^{\prime }}\leftarrow normalized\;labels\;for\;m\;and\;m^{\prime }\;samples$ 7: **end for** 8: $\;W\leftarrow randomly\;initialize\;weights\;for\;{\;\;f}_{\;W}\;layer(s)$ 9: $\;V\leftarrow randomly\;initialize\;weights\;for\;{\;g}_{\;V}\;layer$ 10: **while** notearlystopping **do** 11: $update\;\;W\;\&\;V\;based\;on\;l_{F}\;\&\;l_{R}\;\text{ where,}$ $l_{F}=\sum_{n}\sum_{m\neq m^{\prime }}\left|(\|{\;\;f}_{\;W}({\;x}_{nm})-{\;\;f}_{\;W}({\;x}_{nm^{\prime }}){\|}_{2}-\|{\hat{y}}_{nm}-{\hat{y}}_{nm^{\prime }}{\|}_{2}\right)|$ $l_{R}=\sum_{n}\sum_{m}{\left\|{\;g}_{\;V}({\;\;f}_{\;W}(\;x_{nm}))-y_{nm}\right\|}_{2}^{2}$ $\text{where}\;{\;x}_{nm}\;includes\;ambulatory\;features\;for\;ML\text{-}A\;\;and\;both\;ambulatory\;and\;trait\;features\;for$ ML-AT 12: **end while** 13: **end for**

subsubsectionMetric learning with ambulatory and trait features (ML-AT)

In the second model, ML-AT, of our ablation study, we introduced personalizing our models through additional features as input to the model. These additional features are the trait characteristics (IGTB) (Table 2) features, which were included along with ambulatory features as an input to the SNN architecture of ML-AT. Although the ambulatory features are recorded daily in the dataset, every participant recorded a single value for each IGTB in the overall study (Section 3). So, we duplicated the IGTB features for each input sample of the model per participant in order to implement the model. To avoid biasing the models’ performance and address any data leakage while detecting positive and negative affect, we have removed both trait positive affect and trait negative affect among the trait characteristics during the implementation of both of the detection models. The training process of ML-AT has followed the same steps and number of iterations as the one in ML-A and is also outlined in Algorithm 1.

#### 4.2.2. Separate metric learning models with ambulatory and trait features for each IGTB cluster of participants (ML-C)

**Figure 4:**
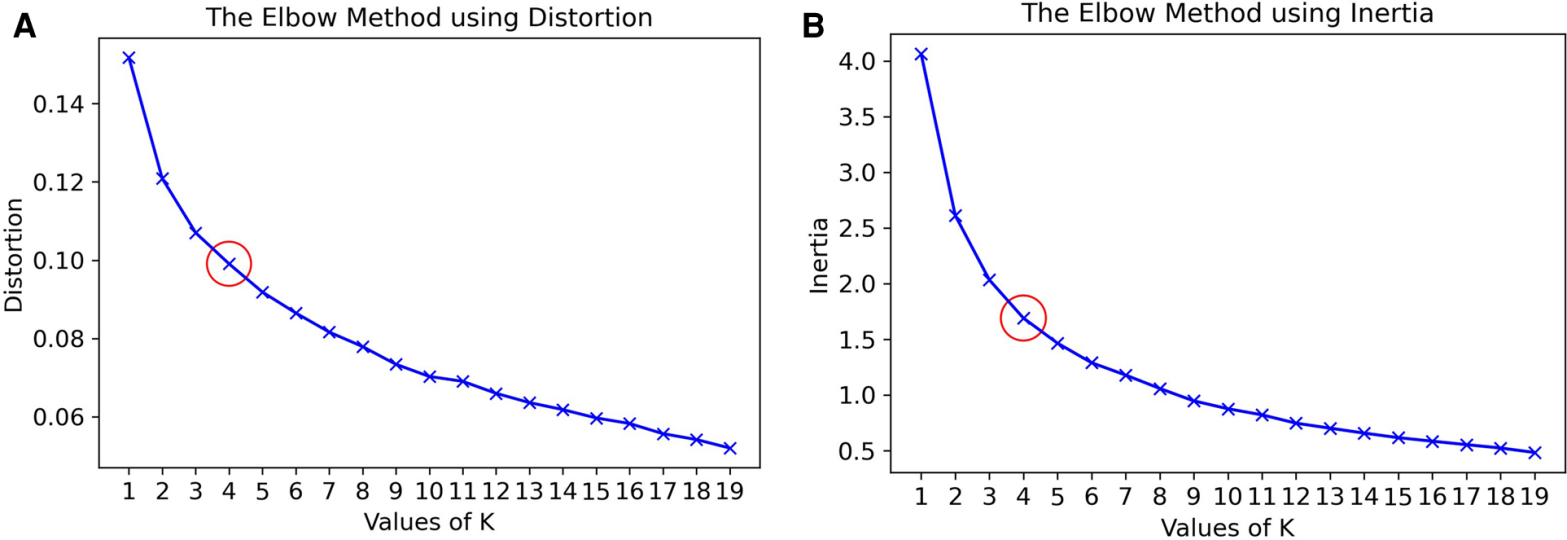
The Elbow method to select value of K in K-means algorithm to for clusters for the Siamese Model for Clustered IGTB. (**A**) Based on distortion and (**B**) based on inertia.

**Figure 5:**
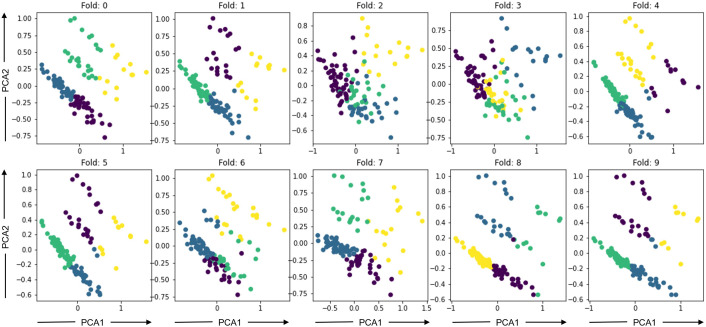
Clusters formed using the K-Means clustering method based on IGTB values where x axis is the principal component 1 (PCA1) and y axis is the principal component 2 (PCA2).

To further explore ways to personalize the model, we developed clusters based on IGTB values recorded by the participants. IGTB questionnaires capture participants’ trait characteristics, therefore this type of clustering stratifies participants based on their psychological characteristics, which are likely to impact the bio-behavioral recordings conditioned on the considered outcomes (36, 77–79). Since the dimension of IGTB is high (14 IGTBs), we reduced the dimension by finding the first 3 principal components of the IGTB feature space through principal component analysis (PCA) (101). Following that, clustering was performed via the K-means algorithm (102), where we decided upon the value of K by optimizing cluster distortion and inertia via the elbow method per (103). Here, *distortion* is the average of the Euclidean squared distance from each sample to the centroid of its assigned cluster, and *inertia* is the sum of squared distances of samples to their closest cluster center. The K value corresponding to the elbow (i.e., “corner”) of the graph is taken as the optimum K, where the distortion and inertia values do not change significantly for higher K values. Since the clustering algorithm does not show a clear elbow pattern in Figure 4 for our dataset, we found the optimal K to be K=4 for our data empirically after experimenting with different K values ranging from 2–4 based on the performance of the validation set. The clusters were formed using only the training data to avoid biasing the output of the model as well as avoiding any data leakage. The K-means model fitted on the training data was used to extract the clusters for validation and test data, and the ML-AT models (Section 4.2.1) were trained on each cluster separately. Figure 5 depicts PCA1 and PCA2 of the 4 clusters in different colors formed using the training dataset developed while iterating through the 10 cross-validation folds. We refer to the new cluster-based models as the separate metric learning models with ambulatory and trait features for each IGTB cluster of participants (ML-C), whose basic algorithm is described in Algorithm 2. The main difference between Algorithms 1 and 2 is, one additional loop is introduced inside the outer loop looping through F. This additional loop loops through the clusters C, and inside the cluster, similar to Algorithm 1, two loops are formed to develop pairs (cluster based in this case) and training the model.

**Algorithm 2:** Algorithm Describing the Separate Metric Learning Models with Ambulatory and Trait Features for Each IGTB Cluster of Participants Model (ML-C).

**Input = folds** (F), **participants** (N), **clusters** (C), **features** (X) **and labels** (Y) **Output = Predicted label** $\left(\bar{Y}\right)$ 1: {Creating folds from participants, one fold for testing, one fold for validation, remaining for training. Samples are taken from the training set. Hyper-parameter tuning and early stopping are done using the validation set. During each for loop for fold, testing is done.} 2: **for**f←1toF **do** 3: forming4clustersbasedonfirst3PCAderivedfromIGTBvaluesofthetrainingdata 4: **for** c←1toC **do** 5: **for** n←1toN **do** 6: $\{{\;x}_{\text{nm}},{\;x}_{\text{nm'}}{\}}_{c}\leftarrow \;formed\;similar/dissimilar\;pairs\;from\;samples\;m\;and\;m^{\prime }(m\neq m^{\prime })\;of\;participant\;n\;from\;cluster\;c$ 7: $\{y_{nm},y_{nm^{\prime }}{\}}_{c}\leftarrow original\;labels\;of\;samples\;m\;and\;m^{\prime }\;of\;participant\;n\;from\;cluster\;c$ 8: $\{{\hat{y}}_{nm},{\hat{y}}_{nm^{\prime }}{\}}_{c}\leftarrow normalized\;labels\;of\;samples\;m\;and\;m^{\prime }\;of\;participant\;n\;from\;cluster\;c$ 9: **end for** 10: ${\;W}_{c}\leftarrow randomly\;initialize\;weights\;for\;{{\;\;f}_{\;W}}_{\;c}layer(s)\;of\;model\;corresponding\;to\;cluster\;c$ 11: ${\;V}_{c}\leftarrow randomly\;initialize\;weights\;for\;{{\;g}_{\;V}}_{\;c}\;layer\;of\;model\;corresponding\;to\;cluster\;c$ 12: **while** notearlystopping **do** 13: $\text{update}\;{\;W}_{c}\;\&\;{\;V}_{c}\;based\;on\;l_{F_{c}}\;\&\;l_{R_{c}}\text{ where,}$ $l_{F_{c}}=\sum_{n}\sum_{m\neq m^{\prime }}|(\|{{\;\;f}_{\;W}}_{\;c}({\;x}_{nm})-{{\;\;f}_{\;W}}_{\;c}({\;x}_{nm^{\prime }}){\|}_{2}-\|{\hat{y}}_{nm}-{\hat{y}}_{nm^{\prime }}{\|}_{2})|$ $l_{R_{c}}=\sum_{n}\sum_{m}\|{g_{\;V}}_{\;c}({{\;\;f}_{\;W}}_{\;c}({\;x}_{nm}))-y_{nm}{\|}_{2}^{2}$ $\text{where}\;{\;x}_{nm}\;includes\;both\;ambulatory\;and\;trait\;features$ 14: **end while** 15: **end for** 16: **end for**

### 4.3. Experimental framework

The SNN, as shown in Figure 3A, has two parts: the shared $f_{W}$ layers and the $g_{V}$ layer. The number of layers in $f_{W}$ is a hyperparameter with values 3–5, each layer containing 64 neurons, where $g_{V}$ contains a single layer with a single node. We used $\ell_{2}$ regularization (regularization value = 0.0001) on each layer and dropout between two layers for the purpose of proper regularization. The dropout value is another hyperparameter in our model with values ranging from 0.1–0.3. To avoid overfitting the model, we utilized early stopping based on the Pearson correlation coefficient of the validation data with patience as a hyperparameter (range: 3, 5, 10). The other hyperparameter we considered is the batch size (128, 256, 512). We optimized our models using stochastic gradient descent (SGD) (104) algorithm.

We used Bayesian optimization using Gaussian processes for hyperparameter tuning. The *gp_minimize* class of the *scikit-optimize* library was used for this purpose. The algorithm was run 200 times to ensure convergence while keeping the function to minimize over the gaussian prior at negative expected improvement. The whole process was repeated for all the folds developed for each of the constructs, keeping the random seed fixed to a constant (constant = 42) for a fair comparison across different runs.

**Figure 6:**
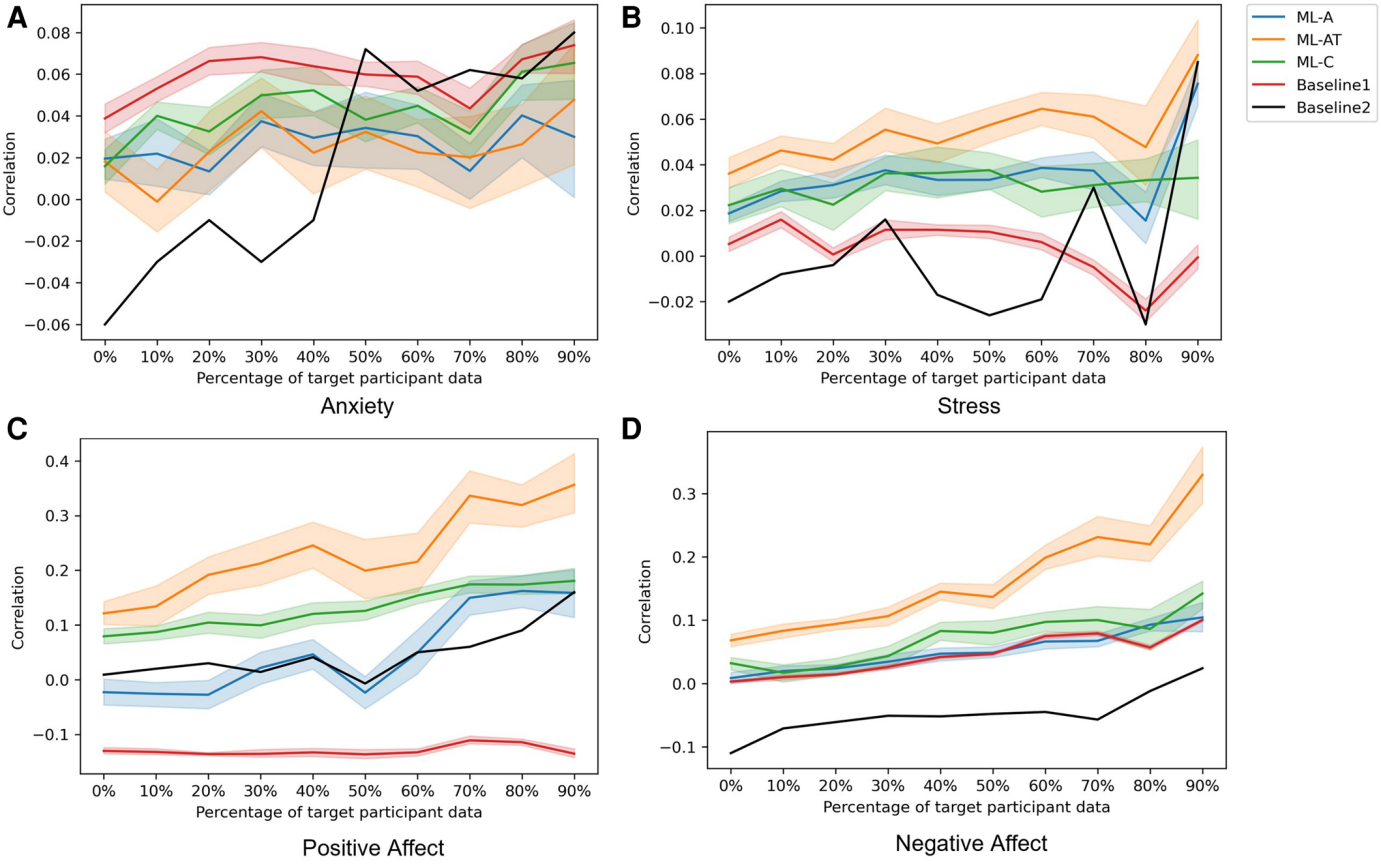
Average Pearson’s correlation coefficient computed over 20 iterations of the considered models, each obtained with a different random restart. The x-axis corresponds to the different percentages of data from the target participants used for fine-tuning the models. The shaded shape around the solid line represents the standard deviation from the 20 iterations. Different colors representing different models as- Orange: ML-AT, Blue: ML-A, Green: ML-C, Red: Baseline1, Black: Baseline2.

After we fixed the participants and tuned the hyperparameters for each fold of the cross-validation loop on a fixed random seed, we saved this information and used it to build models, as well as to develop the train, validation, and test datasets for all the folds of the cross-validation loops separately. We ran these final models 20 times each with non-fixed random seeds ensuring that the model parameters were initialized differently during each run. As a result, we could explore different outcomes of the models for different initialization, thus understanding the overall performance potential of our models. Finally, we report the average of those outcomes and present the average correlation along with the standard deviation of the average value in Figure 6. It is important to note that, we removed the negative affect and positive affect IGTB features while training the ML-AT and ML-C models for the negative affect and positive affect constructs to avoid any data leakage (Section 4.2.1), reducing the number of IGTB features for these two constructs to 12 while producing the Figures 6C,D.

For both ML-A and ML-AT models, we have around 34 thousand pairs for training purposes. The number of similar and dissimilar samples is unbalanced among these pairs. To mitigate the effect of unbalanced pairing while training the models, we weighted the loss function based on the weight of similar and dissimilar pairs of a given cross-validation setting, which gave more weight to the set with fewer pairs. For the ML-C model, we have around 10k pairs for each cluster.

### 4.4. Baseline methods

We used two baseline methods to compare the performance of the SNN model-based ablation study described in Section 4.3. To train and evaluate the baselines, we followed the 10-fold stratified cross-validation method with the same 10 folds used in our proposed methods, following the same participants in each fold, the same hyperparameter for the neural network model construction and training, a similar number of trainable network parameters as the metric learning models (around 12k), and the models were fine-tuned with the same transfer learning technique as used for the proposed models. The ambulatory features were used as the input to the models of the baselines. The first baseline, named Baseline1, uses a feed-forward neural network (FNN), similar to the $g_{V}$ layer of the SNN we propose, except Baseline 1 uses the number of layers equivalent to our proposed SNN models instead of the 1 layer $g_{V}$. This baseline represents a non-personalized model where the ambulatory features serve as the input to the model, instead of the embedding learned through the metric learning process. Our objective in developing such a baseline is to examine whether the learned embedding outperforms the original feature space in predicting different constructs as well as how effective the personalized models are over the non-personalized models in this particular dataset.

As our second baseline, named Baseline2, we utilized the semi-supervised maximum independence domain adaptation (SMIDA) algorithm (105). The SMIDA algorithm proposes a way to reduce the discrepancy between data distribution of different domains. Domain discrepancy arrives in different forms, such as data collected using different devices, in different scenarios. In our case, all the participants’ data were collected using the same types of devices and in a similar scenario. But, inter-individual variation in the data prompted us to design the SMIDA model by considering each participant as a different domain and reducing the domain discrepancy in the feature space through a projection matrix. The algorithm proposes an unsupervised and semi-supervised method of reducing domain discrepancy. We followed the semi-supervised method by keeping the test participants’ data masked while learning the projection matrix. Among the kernels developed for creating the projection matrix, we opted for the linear one in our experiments. The projected matrix has been used as input to a linear regression model that estimated the MGT output.

## 5. Results

Although our highly skewed dataset is very suitable for classification experiments (Figure 1), instead of forming a classification problem from our dataset, we focused on doing a regression analysis to predict the original labels through our model. Because performing regression analysis on the constructs has given us the ability to avoid artificially discretizing the predicted labels. Moreover, this improves the resolution of detection of the constructs thus making the models better suitable for designing JITAIs. We have used Pearson’s correlation coefficient to evaluate our proposed models and baselines. In this section, we report the average and standard deviation of Pearson’s correlation coefficient between the original and predicted MGTs across 20 iterations (i.e., different random initializations of the models) when using an increasing percentage of samples from the target participants for fine-tuning (Figure 6). Different colors in Figure 6 represent the different models described in Section 4. According to our findings, the ML-AT model works the best in all the constructs we examined except for anxiety. This finding demonstrates that introducing trait information helps in improving the performance of the models and yields better accuracy when estimating daily constructs. Trait characteristics were not employed as sole predictors, because our goal is to estimate participants’ states at the daily level. This requires behavioral information recorded via physiological and acoustic signals, allowing to capture of momentary fluctuations that cannot be recorded via the trait scores. Although our proposed models do not outperform baselines while predicting anxiety, the performance of the models is not significantly lower compared to the baseline methods for anxiety (Table 3). Apart from positive affect, all other constructs have severely skewed distribution (Figure 1), which might also affect the overall performance of the models. The second-best performing model for positive and negative affect is the ML-C (Figures 6C,D), while ML-C and ML-A perform similarly in the case of stress (Figure 6B). We have performed experiments keeping all 14 IGTB features for the positive and negative affect, which produces similar graphs as in Figures 6C,D. We further observe an increasing trend in performance for all the models when using more data from the target participants for fine-tuning. Such a trend is expected as models trained using a portion of the data from the target participants learn their unique distribution, thus performing better in the test samples. The varying performance of the clustering algorithm (i.e., some clusters are better separated in some folds compared to other folds in Figure 5) appears to affect the overall performance of the ML-C model. For example, the Pearson’s correlation coefficient for the positive affect construct for fold 1, which has separable clusters, is 0.28(p<0.001), while the same value decreases to 0.04(p>0.05) for fold 3 with less separable clusters.

**Table 3:** Results from one-tailed paired-sample t-test evaluating significant differences between the predicted values from ML-AT, ML-A and Baseline 1 in the form of average Pearson’s correlation coefficient over 20 iterations between true momentary ground truth (MGT) and predicted values of different constructs utilizing different percentage (%) of target data for fine tuning purpose.

MGT	Percentage (%)	ML-AT vs ML-A	ML-AT vs Baseline1
	0	t(19)=−0.15, p>0.05	t(19)=−2.5, p>0.05
Anxiety	40	t(19)=−0.6, p>0.05	t(19)=−3.79, p<0.001
	90	t(19)=0.86, p>0.05	t(19)=−1.6, p>0.05
	0	t(19)=4.33, p<0.001	t(19)=8.89, p<0.001
Stress	40	t(19)=2.72, p<0.001	t(19)=8.36, p<0.001
	90	t(19)=1.32, p>0.05	t(19)=10.44, p<0.001
Positive effect	0	t(19)=8.6, p<0.001	t(19)=21.5, p<0.001
	40	t(19)=7.61, p<0.001	t(19)=16.74, p<0.001
	90	t(19)=5.39, p<0.001	t(19)=17.42, p<0.001
Negative effect	0	t(19)=8.95, p<0.001	t(19)=13.27, p<0.001
	40	t(19)=11.58, p<0.001	t(19)=15.38, p<0.001
	90	t(19)=8.54, p<0.001	t(19)=9.76, p<0.001

Next, we perform one-tailed paired-sample t-tests between the ML-AT, ML-A, and ML-AT, Baseline1, to examine the statistical significance between the proposed approaches and the baseline methods (Table 3). Results are reported for three different percentages of samples from target participants in the training data (i.e., 0%, 40%, and 90%). The ML-AT model’s performance for stress, negative affect, and positive affect is significantly higher (p<0.05) compared to the ML-A and Baseline1 models. Baseline1 performs the best for anxiety, although the performance among different models becomes similar as we increase the percentage of data taken from our test dataset for fine-tuning the model.

Through our proposed metric learning approach, we train the network by learning the relative distance between features with respect to the behavioral constructs of interest. In this way, the embeddings learned through this process should be able to reliably estimate the behavioral constructs (Figure 6, Table 3), while they should not retain person-specific information. To test this hypothesis, we have used the original features and the embeddings learned by the ML-A to classify the different participants of the dataset. We have utilized a logistic regression (LR) model for this person identification task and have considered embeddings learned from the different percentages of samples from the target participant used for fine-tuning. The average accuracy is calculated across 20 iterations that correspond to random initializations of the LR models (Table 4). The embedding features learned by the ML-A model appear to retain less participant-dependent information compared to the original features, as reflected in the lower participant classification accuracies of the first model (e.g., 41.92%) compared to the second (e.g., 83.21%). The fact that the embeddings learned by the ML-A models depict reduced variability across participants might also be a potential reason why the proposed metric learning approaches outperform conventional models, which might not be able to fully disentangle participant-dependent and construct-dependent information.

**Table 4:** Person identification accuracy in percentage (%) over 20 iterations using original features and embedding learnt from the Siamese Model (ML-A) for different percentage of fine tuning data from different momentary ground truths (MGT).

MGT	Features	0%	10%	20%	30%	40%	50%	60%	70%	80%	90%
Anxiety	Original	83.21	81.28	79.66	77.96	76.11	74.61	71.29	60.78	53.93	15.86
	Embedded	41.92	38.71	36.44	35.06	30.79	28.63	27.13	18.86	17.75	10.06
Stress	Original	83.15	80.96	79.60	77.18	76.99	73.93	67.68	58.96	49.59	17.20
	Embedded	53.31	50.83	48.28	47.46	44.06	40.59	37.97	28.60	25.09	11.22
Positive effect	Original	83.11	81.08	79.71	76.98	74.92	73.55	69.78	57.40	48.03	15.85
	Embedded	47.10	45.03	42.16	41.86	37.59	35.05	32.71	25.40	24.17	13.58
Negative effect	Original	83.11	81.34	79.25	77.85	76.08	73.49	68.65	60.38	47.08	15.76
	Embedded	51.79	48.64	48.12	46.09	42.98	38.31	37.64	30.22	26.22	14.29

**Figure 7:**
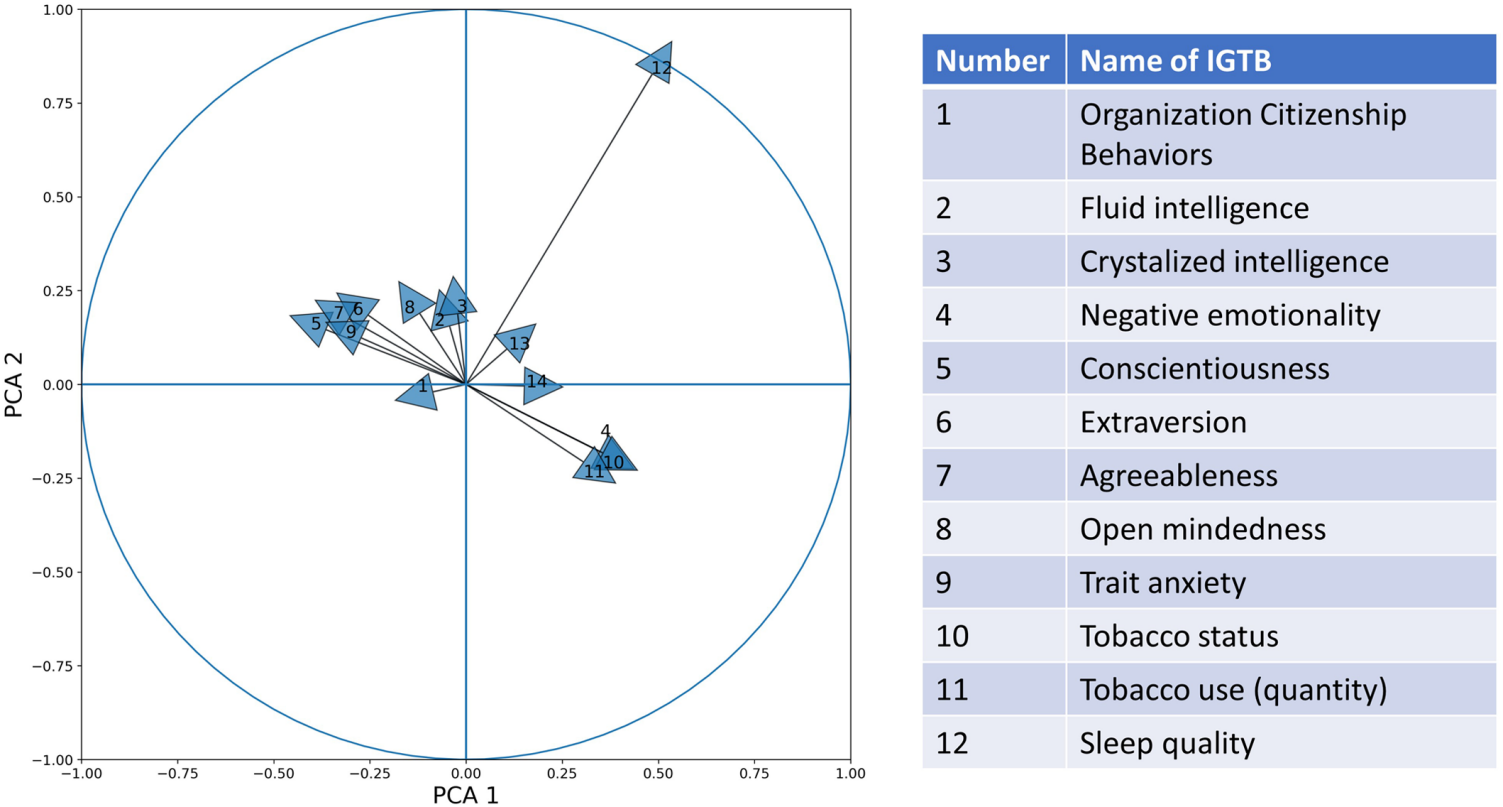
Correlation circle plot with a unit circle for PCA 1 and PCA 2 using different IGTB values.

We explored the effect of the different IGTBs on constructing the PCA dimensions. For this purpose, we plotted a correlation circle plot showing the correlation between different IGTB values with the first two PCA dimensions (Figure 7). PCA 1 and PCA 2 explain 31.96% and 16.61% of the total variance of the data, respectively. The large inter-individual differences played a significant role in reducing the amount of variability explained by the first two principal components. Through the unit circle, it is easy to compare the effect of different IGTBs on developing the PCA dimensions. From the figure, we can find that Tobacco use (Yes/ No), which reflects whether the person uses tobacco or not, has the largest contribution to building the PCA dimensions, but contributes almost equally to both the first and second PCA components. Fluid intelligence, crystallized intelligence, and openness contribute largely toward the second PCA dimension, while sleep quality contributes mostly to the first. Trait negative affect, trait anxiety, and emotional stability form a cluster contributing positively to the first PCA component and negatively to the second. On the other hand, extraversion, agreeableness, trait positive affect, organization citizenship behavior, form a different cluster contributing positively to the second PCA component and negatively to the first one.

In inspecting those clusters in more detail, Table 5 further shows the mean of the normalized IGTB values computed for the participants of each cluster. Analysis of Variance (ANOVA), along with the Kruskal-Wallis test, indicates that all IGTB measures are significantly different across these four clusters (cluster 0 with 1651 samples, cluster 1 with 368 samples, cluster 2 with 584 samples, and cluster 3 with 997 samples). Upon close examination of this table, we can provide an additional description of the characteristics of the participants in each cluster. Since the Tobacco quantity value is zero in clusters 0 and 3, the *t*-test statistics in N/A in this case. Cluster 0 includes participants who have scored the highest on organization citizenship behaviors, conscientiousness, extraversion, agreeableness, openness, and positive affect. On the contrary, the cluster shows the lowest mean score for fluid intelligence, emotional stability, negative affect, trait anxiety, Tobacco use (Yes/ No) and use, and sleep quality. We note here that the lower the score for sleep quality, the better the overall sleep is. Thus, this cluster contains participants who do not use tobacco, have good sleep quality, have low anxiety, and are socially extroverted, highly agreeable, and open-minded. On the contrary, individuals assigned to cluster 1 appear to have opposite traits compared to the ones assigned to cluster 0 with the corresponding participants depicting the lowest values in organization citizenship behaviors, conscientiousness, extraversion, agreeableness, and positive affect; and the highest values in both fluid and crystallized intelligence, emotional stability, negative affect, trait anxiety, Tobacco use (Yes/No), and sleep quality. These suggest that cluster 1 includes participants with high intelligence, but lower overall well-being and higher negative traits. Clusters 2 and 3 appear to be “in the middle.” Cluster 2 is more closely related to cluster 0. Individuals in cluster 2 have some of the lowest values for emotional stability and trait anxiety. These constructs are not significantly different between clusters 0 and 2 based on the post-hoc *t*-test analysis. Similarly, with the exception of the low values for crystallized intelligence and tobacco use, cluster 3 is more closely related to cluster 1. Individuals belonging to this cluster scored the lowest on openness and high on crystallized intelligence, emotional stability, and trait anxiety. Summarizing our findings, we can see that cluster 0 and cluster 2 are similar and include individuals with positive affectivity who tend to keep a relatively healthy lifestyle in terms of sleep and tobacco use. On the other hand, cluster 1 and cluster 3 are similar in that they include individuals with negative affectivity and relatively less healthy lifestyle.

**Table 5:** Mean of normalized IGTB values across four clusters with ANOVA, Kruskal-Wallis, and t-test between clusters. Since most values are statistically significant, only non-significant values are shown using an asterisk.

Name of IGTB	Cluster				Statistics value							
	0	1	2	3	$F(3,3596{)}^{a}$	$F(3,3596{)}^{b}$	T-test between clusters					
							0 vs 1	0 vs 2	0 vs 3	1 vs 2	1 vs 3	2 vs 3
							t(2018)	t(2234)	t(2647)	t(951)	t(1364)	t(1580)
Organization citizenship behaviors	0.53	0.36	0.47	0.43	107.8	205.83	17.9	5.98	11.76	−8.87	−6.99	3.15
Fluid intelligence	0.58	0.62	0.59	0.63	9.56	49.61	−3.21	−2.37	−6.41	1.80${}^{*}$	−0.61${}^{*}$	−3.70
Crystallized intelligence	0.62	0.66	0.62	0.57	6.03	7.67	−3.19	0.30${}^{*}$	6.03	2.89	6.84	4.20
Emotional stability	0.24	0.66	0.30	0.48	1164.29	877.31	−50.84	−6.67	−44.30	36.30	22.69	−22.81
Conscientiousness	0.82	0.36	0.73	0.55	1363.63	973.16	53.78	12.51	35.38	−36.01	−18.21	18.88
Extraversion	0.65	0.35	0.63	0.41	422.14	621.88	31.84	2.34	36.34	−25.24	−6.25	24.97
Agreeableness	0.74	0.42	0.65	0.52	642.54	737.06	40.55	10.72	29.60	−24.14	−10.52	14.50
Openness	0.67	0.62	0.65	0.52	14.32	37.86	5.23	2.16	19.77	−3.12	8.13	13.96
Trait positive affect	0.70	0.41	0.59	0.50	416.55	604.60	30.35	13.83	27.14	−18.15	−8.64	11.82
Trait negative affect	0.15	0.59	0.16	0.33	1370.23	742.50	−32.42	−2.55	−25.84	29.87	17.98	−20.08
Trait anxiety	0.21	0.55	0.21	0.42	1078.97	798.20	−36.07	0.46${}^{*}$	−41.96	33.66	13.69	−33.52
Tobacco use (Yes/ No)	0	0.80	0.70	0.03	7399.80	2477.45	−61.42	−68.68	−7.25	6.11	57.43	62.59
Tobacco quantity	0	0.11	0.04	0	188.74	921.30	−9.08	−9.58	N/A	5.16	9.08	9.58
Sleep quality	0.22	0.38	0.25	0.30	148.56	179.32	−13.10	−4.24	−10.54	9.99	6.86	−5.09

^a^ANOVA

^b^Kruskal-Wallis

${}^{*}p>0.05$ (not significant)

## 6. Discussion

In this work, we have examined the effectiveness of metric learning algorithms in learning behavioral outcomes from ambulatory data, potentially addressing the inherent inter-individual variability that tends to hamper model performance. The performance of our proposed model has been evaluated on ambulatory data collected in an uncontrolled environment with respect to several constructs related to the mental and emotional well-being of healthcare workers, such as affect, stress, and anxiety. We have achieved personalization by utilizing the trait characteristics of the participants as an additional input to the models as well as clustering criteria for grouping the participants, followed by training separate metric learning models for each group. Personalization implemented by adding the IGTB features as an additional input to the model (i.e., ML-AT) outperforms the ML-A models that only include the ambulatory-based features suggesting the need for adding information about individuals’ trait characteristics in the model. Moreover, our results have demonstrated that fine-tuning our model using a portion of data from the target participants can improve the model performance rather than training the model solely on samples from participants other than the target. We have also explored the role of different IGTBs on the resulting participant cluster. This provides us an additional intuition on the key participant traits to focus on in order to develop group-based models.

Studies among healthcare workers have shown that improvement of the overall workplace environment is possible through proper mindful interventions (106, 107). Healthcare workers are becoming more aware of their health, safety, and wellness, while issues associated with managing stress and promoting overall well-being (e.g., weight control) are becoming more and more pressing. Technology tools, such as intelligent ambulatory assessment via personalized models, could help in assisting healthcare workers for the same purpose. Prior work has found that healthcare workers view positively the integration of such smartphone-based technologies in their daily activities which could potentially lead to improving their well-being and mitigate the risk of depression (108), thus similar personalized machine learning models could be used as a foundation of such smartphone apps. The proposed metric learning model is quite lightweight (i.e., around only 12K trainable parameters) compared to the state-of-the-art convolution neural network (CNN) for mobile applications, MobileNetV3’s 2.9 million parameters (109) and can utilize a small amount of collected data for training purposes, rendering it suitable for lightweight app development of momentary psychological evaluation and intervention. This alternate way to assess the mental condition of healthcare workers has further the potential to yield higher adherence rates compared to conventionally-used self-reporting via ecological momentary assessment (EMA) since it only requires participants to wear their wearable devices and have their smartphone devices in close proximity.

Our proposed SNN and personalization techniques demonstrate improvements over methods that do not account for individual differences, however, the performance is yet insufficient for creating effective JITAIs. Utilizing a dataset collected in a real environment with multi-modal features has posed a unique challenge. Further, we have formulated the task as a regression problem, where the commonly used matrix related to tasks predicting similar constructs are accuracy and the F1 score (110–114). A recent review of studies classifying stress demonstrates that the overall accuracy of detecting stress through data collected from ambulatory devices (around 75%) is significantly lower than utilizing data collected in a laboratory setup (around 95%) (115, 116). From the distribution of the labels of different constructs (Figure 1) we observe that, apart from positive affect, all the other constructs have a severely skewed distribution. Especially, for anxiety, more than 50% data belongs to label 1, and for negative affect, almost 80% of the data belongs to label 5. This might be the underlying factor for the relatively better performance for predicting positive affect than the other constructs using our proposed models. In comparing our results with prior work, Pearson’s correlation coefficients in estimating anxiety from ambulatory data were found to be similar to ours (cross-validated R2 of 0.06, permutation test p<0.001) (117). Although Yan et al. (66) utilized the TILES-2018 dataset to predict the affective state of the participants via regression analysis, the method of personalization carried out in this work varies since the authors used data from participant’s prior week’s to predict the affect in future weeks. Moreover, the individual-specific models, trained using rescaled and transformed features based on individual-specific statistics (i.e. average and standard deviation) contrast significantly with our fold-wise cross-validation approach. Finally, along with the factors discussed above, the usage of contextual features and a relatively larger dataset (i.e., a combination of TILES-2018 and 2019, with 10-weeks data per participant as opposed to on average 3 weeks of data used in our study) could explain the overall better performance of this study. Overall, there is a shortage of prior work in ambulatory monitoring that uses similar evaluation metrics for our considered behavioral constructs. Another potential reason for the relatively low performance of our models lies in the fact that the self-reported labels used to train these models might not necessarily be collected in close temporal proximity to the occurrence of the daily event that led to the corresponding value at the self-report. For instance, a participant might have reported negative affect at the end of the day, but this report might have reflected an event that happened many hours before the self-report questionnaire was administered. The proximity between event and self-report could significantly impact the overall performance of the models. For example, using data collected in the wild from 606 individuals (67) achieved a 0.25 Spearman’s correlation for stress detection. Apart from the fact that the dataset is relatively larger than the dataset we used in this study, this data also ensured that the participants would respond to the self-reports in close proximity to the event. This could potentially help in achieving relatively better performance. This lack of coordination between the event and the label could be potentially addressed via saliency detection methods, which will allow us to explore prominent temporal patterns in the data that might be indicative of changes in behavioral constructs.

Despite the encouraging results, our study presents the following limitation. So far, we have studied well-being measures that are aggregated over an entire day. As part of our future work, we will explore the dataset in finer time resolution than predicting our target constructs on a daily level, which will be the next step in developing an effective JITAI using our proposed approach. Our current MGT labels are recorded once daily limiting our ability to use the data in achieving this objective. Moreover, exploring patterns in data across a day instead of using the daily average value for predicting the constructs would help in pinpointing specific patterns which may trigger stress, anxiety, or affect a person both positively or negatively, thus improving upon our current findings. Another limitation of this study is the pre-defined similarity threshold between samples in our proposed metric learning approach. Determining the appropriate value of the similarity threshold, potentially for each participant separately, could have further improved our results and revealed additional insights regarding individual differences in our data. This study is also limited by a small dataset. To address this limitation, we plan to expand our current study in a cross-corpus manner extending to other publicly available datasets in this or similar domain, for example, TILES-2019 (65), Tesserae (118), and publicly available multimodal dataset on stress detection of nurses (119). Our results indicate that eliminating individual-specific differences from the data via metric learning can yield improved performance in detecting well-being outcomes. This can inspire the use of differential privacy approaches (e.g., using adversarial learning), which can potentially further eliminate individual-dependent attributes from our data and preserve, or even further attenuate, the behavioral attributes of interest.

## 7. Conclusion

Mental well-being is a crucial part of overall well-being and effective work performance. This paper examines a machine learning algorithm based on metric learning that contributes to assessing one’s mental and emotional well-being unobtrusively through widely-available ambulatory sensing technology. We have proposed a lightweight model which utilizes a metric learning technique to learn the relative distance between similar and dissimilar samples and thereby reduce person-specific signal dependencies in the data. We have bolstered the personalization of our proposed approach by utilizing the participants’ trait characteristics as added features to the model and criteria for clustering the population of the dataset to form cluster-specific models. The performance of the proposed models has been compared against two baselines, one that has utilized a non-personalized learning technique via a model trained on all participants, and one that has integrated personalization via a domain adaptation technique rather than metric learning. Results from our experiments indicate that our proposed models outperform the baselines in most cases. Among the proposed approaches, models utilizing IGTB features perform better than those not using the IGTB features, highlighting the need for personalization. The proposed system paves the way for developing in-the-moment interventions that can promote a healthier workplace environment for healthcare workers and other professionals who work in similar high-paced high-stakes environments.

## Data Availability

Publicly available datasets were analyzed in this study. This data can be found here: https://tiles-data.isi.edu/homepage.
